# Treatment thresholds and minimal clinically important effect sizes of antiosteoporotic medication–Survey among physicians and lay persons in Germany

**DOI:** 10.1371/journal.pone.0272985

**Published:** 2022-08-11

**Authors:** Piet van der Keylen, Nikoletta Zeschick, Anna Ruth Schlenz, Thomas Kühlein

**Affiliations:** Institute of General Practice, Friedrich-Alexander-University Erlangen-Nürnberg, Erlangen, Germany; Universiti Putra Malaysia, MALAYSIA

## Abstract

**Background:**

Patient decisions to take preventative treatments for osteoporosis depend on their perceptions of fracture risk, medication effect sizes (ES) of benefits and harms. However, physicians and lay persons may have differing perceptions of risks and medication efficacy. Both tend to overestimate medication benefits. This study surveyed at what risk physicians would prescribe and lay persons would be willing to take bisphosphonates, the minimum ES both groups do demand and the physicians estimates of the actual benefit of bisphosphonates.

**Design:**

Cross-sectional online questionnaire survey.

**Methods:**

Respondents were confronted with a case vignette with an osteoporotic patient (10-year femoral fracture risk: 32%). They were asked at what threshold of 10-year-risk of femoral neck fracture they would prescribe or take a drug. They were asked for the minimum ES (absolute risk reduction, ARR) they demand from bisphosphonates to prescribe or take them. Physicians were asked to provide their estimate of the actual ARR of bisphosphonates.

**Results:**

114 physicians and 140 lay persons answered (convenience sample/snowball distribution). The 10-year-risk threshold of lay persons (*Mdn*_lay_ = 60%) willing to take medication was twice as high as the physicians’ threshold (*Mdn*_phy_ = 30%) to prescribe it (*p* < .001). The median minimum ARR physicians demanded for bisphosphonates prescription was 17%, whereas lay persons demanded 22% (*p* < .001). Physicians estimated the actual ARR of bisphosphonates to be 12%. This estimated effect size was below their own minimum threshold for prescription.

**Conclusions:**

Lay persons tolerate a higher fracture risk and demand a larger benefit of antiosteoporotic medication for fracture prevention than physicians. Physicians demand higher minimum benefits than their own estimates which in turn are above the benefit evidence suggests. Physicians should be more familiar with ES of antiosteoporotic drugs concerning patient outcomes and actively advise lay persons before preventive treatment decisions are taken.

## Background

Osteoporosis is defined as a T-score for bone mineral density (BMD) of ≤ -2.5 standard deviations (*SD*) below the mean of young female adults assessed by dual-energy x-ray absorptiometry (DXA) [1–3]. It is conceptualized as *“a systemic skeletal disease characterized by low bone mass and microarchitectural deterioration of bone tissue”* [4]. As bone-density declines with age this operational definition generates an osteoporosis prevalence of about 50% within female populations 80 years and older [2]. While osteoporosis itself does not cause direct suffering for patients, the disease burden itself arises from occurring fractures [5]. The individual risk for a fracture can be calculated by different risk assessment tools like the country specific *Fracture Risk Assessment Tool* (FRAX®) that calculates an individual 10-year risk for *major fractures* [6]. The guideline for German speaking countries (DVO, Umbrella Organization of German-Speaking Scientific Osteological Societies) uses a different method to calculate risk for fracture (DVO risk model) [1]. The threshold of the DVO to start specific antiosteoporotic treatment is set at a 10-year risk of ≥ 30% for proximal femoral and vertebral fractures or ≥ 14% for any major osteoporotic fracture (defined as clinical vertebral, hip, forearm, or proximal humerus fracture) [1]. The *“U*.*S*. *National Osteoporosis Foundation Clinician’s Guide to Prevention and Treatment of Osteoporosis”* (NOF) sets a 10-year risk threshold for any major osteoporosis-related fracture for pharmacological intervention of ≥ 20% and ≥ 3% for hip fractures [7]. The NOF criteria have been applied to a data-set of US-American women estimating that that at least 72% of U.S. white women ≥ 65 years of age and 93% of those ≥ 75 years of age would be recommended for drug treatment [8]. We estimate that applying the DVO criteria would make at least about 75% of women ≥ 75 years in Germany eligible for taking antiosteoporotic medication.

While such models recommended by guidelines present a feasible approach for general risk and treatment assessment, the question remains, if guidelines sufficiently communicate information about effect sizes (ES) of harms and benefits at an individual level [9]. Comprehensible and applicable ES are especially important, as communication of preventive measures depends on how the subject at risk and the physician perceive the individual risk of fractures and the ES of preventive medication to reduce this very risk [10, 11]. Studies indicate that patients often set treatment thresholds differently and also assess benefits and harms differently than their physicians [12–14]. Generally, evidence shows that physicians and patients overestimate benefits and underestimate harms of drugs [15–17].

One group of drugs for specific antiosteoporotic therapy are bisphosphonates [2], with the two most commonly used being alendronate and risedronate [18, 19]. In a best estimate scenario two Cochrane meta-analyses of randomized-controlled trials (RCTs) communicated specific ES for these drugs showing an absolute risk reduction (ARR) of 1.0% (i.e. number needed to treat (NNT) 100 over three years) in the secondary prevention of hip fracture with little or no effect in primary prevention [20, 21]. A more recent systematic review identified an ARR of .57% for hip fracture over three years, resulting in a NNT of 175 [22]. Evidence is weak for women older than 75, as only a limited number of studies have included enough patients in this age group. In some studies, significant effects on hip fracture in this group could not be shown [23–25]. Evidence from RCTs in elderly men that contribute around 30% to 40% of hip fractures [26] is even more sparse.

Statistical significance seems to notoriously be confused with clinical relevance. Statistical significance is a function of sample size and it has been proposed to retire it as a meaningful statistical concept [27]. Instead, the concept of a *minimal clinically important difference* (MCID) seems a more meaningful approach. It defines how much an outcome would have to change minimally in order to be relevant for the individual patient [28]. According to the paradigm of evidence-based medicine (EBM), treatment decisions ultimately are subjective, depending on individuals preferences and clinical judgement ideally in face of the currently best available evidence [29]. As preferences and judgements might also be culture, country or ethnic-specific, or based on personal experience [30–32] we wanted to add to the still sparse evidence that current guideline recommendations for osteoporosis and physician perceptions might not meet MCID of lay persons in Germany. To our knowledge all guidelines conflate some or all major fracture types to the concept of “major osteoporotic fracture” for their risk prediction models and for the therapeutic thresholds they set. As the fracture types are different in their frequencies, the ages they happen, the mechanisms they are based on and their consequences for the patients, we perceive this conflation as inappropriate for a meaningful decision making in the individual. Therefore, we chose focusing on the most severe type of fracture in terms of consequences for the patient, namely femoral fractures.

### Aim of the study

Our aim was to survey the minimum threshold of 10-year risk for femoral neck fracture at which physicians would prescribe bisphosphonates and lay persons would be willing to take them (treatment threshold). Additionally, we wanted to know the demanded minimum clinically important ARR (minimum ES in the sense of MCID) by each group in order to prescribe or take bisphosphonates, respectively. Lastly, we wanted to learn about the estimated real benefit (estimated ARR) of bisphosphonates among physicians.

## Material & methods

### Design

A quantitative, cross-sectional, online questionnaire study was conducted, following the STROBE guideline and CHERRIES checklist [33, 34]. Both checklists and the items relevant for the present study and their fulfilment can be found in **S1** and **S2 Appendices.** The study has been approved by the Ethics Committee of the Friedrich-Alexander University Erlangen-Nürnberg (no. 145_19B). The software SurveyMonkey® was used for programming and developing the questionnaires and subsequent online rollout and anonymous data collection [35]. Participants gave informed consent accepting privacy regulation of the survey software presented at the begin of the survey. Questionnaires for physicians and lay persons were developed at the Institute of General Practice of the Friedrich-Alexander-Universität Erlangen-Nürnberg based on opinions of experts in general practice and educational research. Consensus was reached after discussion and referring to similar work [12]. Pre-testing for design and content evaluation was done with four physicians and four lay persons using the *“thinking aloud”* method [36]. Changes in question sequence to reduce priming effects in questions to risk reduction and treatment thresholds were made due to pre-test analysis.

A voluntary, convenience sample was collected by sending the link to the questionnaire via email to physicians and lay persons. Inclusion criteria were being a physician, with or without completed specialty training, or being a lay person older than 18 years. The questionnaire contained a description of the study’s objective, informed consent statement, data handling (anonymity) and invitation to participation. The email was delivered between November 2019 and February 2020. Physicians of the institution’s internal educational network (KWAB, Competency Education Center in General Medicine) and physicians receiving the newsletter of the Bavarian Association of General Practitioners (BHÄV) were invited to participate and asked to forward the link to colleagues in a snowball system. Lay persons were invited on a basis of personal contacts and networks by sending the link via email, asking them for snowball distribution of the link. A response rate therefore cannot be reported. No reminder was sent. There was no advertising of the study and no incentives for participation were offered. In order to obtain an effect of d = .5 in a Mann-Whitney-Wilcoxon test for two independent samples and a two-sided α-error level of .05 with a power of .95 a sample size of 110 participants per group was calculated [37]. Data collection was closed, when the aimed sample size was reached.

Questionnaires were presented in a (non-randomized, non-adaptive) scrollable web-page consisting of eight questions for the physician group and six questions for the lay person group. At the end a completeness check was offered, also indicating possible wrong or missing entries by the user.

#### Information about osteoporosis

**Fig 1:**
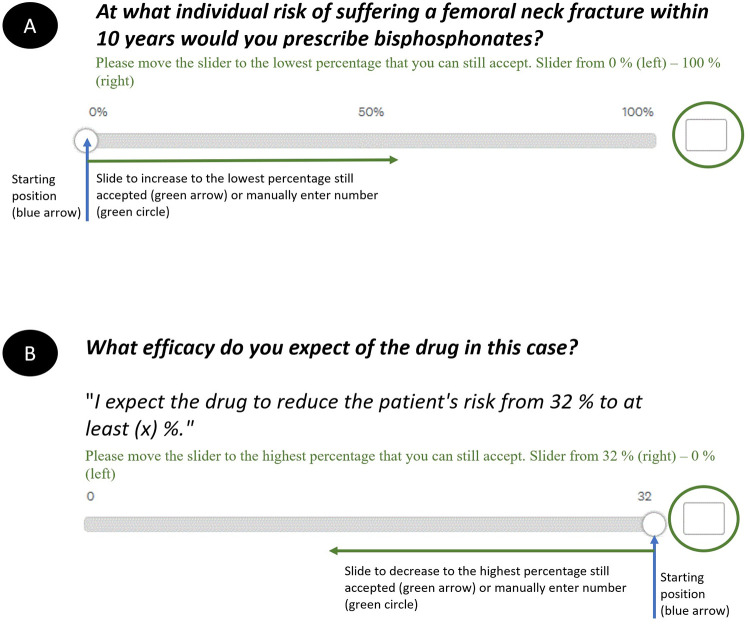
Exemplary depiction of the physicians’ survey to display treatment threshold (A) and demanded minimum ARR (B). The different starting positions (blue arrows) and the different sliding directions (green arrows) in order to actively increase/decrease risk value. Manual entry was able by entering numbers in the text field (green circles).

Both groups were briefly informed about osteoporosis. Treatment options for reducing fracture risk with bisphosphonates were explained. Possible side effects and their frequencies were listed according to the German product information for alendronate. As lay persons might not be fully aware of the medical definition and complications of femoral neck fracture, they additionally received comprehensible information about femoral neck fractures and possible clinical implications. In pre-testing of lay persons, we were mindful on basis of this given information, that lay persons felt adequately informed on osteoporosis and femoral neck fracture. Additionally, with femoral neck fracture being common among elderly patients, we estimated that a majority of lay persons had come into contact to osteoporosis or resulting fracture in their environment. Physicians then received four, lay persons three questions in the same sequence in their role as physician or patient in order to *“prescribe bisphosphonates”* or “*take bisphosphonates*”, respectively. As relative risk numbers often lead both physicians and patients to misjudgment and in line with prior work by Steel and Douglas et al. [12, 13] the treatment threshold was asked in absolute risk numbers [38]. **Fig 1** exemplary shows two questions for physicians. The whole questionnaire can be accessed in **S3 Appendix.**

### Measures

#### Sociodemographic characteristics

To analyze possible subgroup effects and to sufficiently describe the sample, physicians were asked for gender, age, specialty and occupational status. Lay persons were asked for: gender, age and educational status. The question for educational status was introduced as evidence has shown that a lower level of education may be associated with higher minimum benefit to justify intervention use [16]. Educational status of lay persons was collected based on categories by the German Federal Office of Statistics [39].

#### Treatment threshold

The question posed was:

**“***At what individual risk of suffering a femoral neck fracture within 10 years would you* ***prescribe*** *(physicians)* ***OR*** ***take*** *(lay persons) bisphosphonates*? Please move the slider to the lowest percentage that you can still accept. Slider from 0% (left)– 100% (right)”; 1% steps possible; free text field on the right for manual number entry. Starting position of the slider set at 0% (left) to actively increase to the lowest absolute risk percentage still accepted, by sliding to the right.

#### Demanded minimum ARR

A patient scenario was presented and slightly adapted to both groups. We presented a 78-year-old female patient with no prior fractures, with a 10-year fracture risk of slightly more than 30%, but no additional information. Since evidence is weak for this age group, we wanted to create an “inconvenient” and “grey” patient scenario, where no clear “right” or “wrong” lies obviously at hand, creating ambiguity in the physicians’ decision, not only relying on external evidence, but experience and personal preference, therefore practicing the very definition of EBM according to Sackett et al. [29].

*“You (lay persons)/Your patient (physicians) was diagnosed with osteoporosis by the age of 78*. *She has not yet suffered a previous fracture due to it*. *Please assume*, *your/your patient’s 10-year risk of femoral fracture is 32% without medication*. *This means that 32 out of 100 similar patients will suffer a femoral neck fracture within the next 10 years*. *There is*, *however the chance to reduce your/your patient’s risk by medication like bisphosphonates*. *What efficacy do you expect of the drug in this case*?”

"*I expect the drug to reduce the patient’s risk from 32% to at least x%*.*"* (Please move the slider to the highest percentage that you can still accept. Slider from 0% (left)– 32% (right); 1% steps possible; free text field on the right for manual number entry. Starting position of the slider set at 32% (right) to actively decrease to the desired, lower risk by sliding to the left.)

#### Estimated ARR

Only physicians were asked, how they estimate the actual ES of bisphosphonates for that patient by presenting further information: T-Score: -3.0, BMI: 25.7, no other risk factors.

*"The patient’s risk of suffering a femoral neck fracture within 10 years is reduced by regularly taking a bisphosphonate from 32% to x%*.*"* (Please move the slider to the estimated percentage. (Slider from 0% (left)– 32% (right); 1% steps possible; free text field on the right for manual number entry).

#### Recent fracture

Both groups were asked

*“Did you suffer a fracture within the past 12 months*?”

### Data analysis

The software IBM^®^ SPSS^®^ Statistics 24 was used for statistical processing and data analysis. Data from both groups were not normally distributed (Kolmogorov-Smirnov *p* < .001). Therefore, non-parametric methods were used for data analysis. A Mann-Whitney U-test was used to compare the independent groups of physicians and lay persons. To compare the *demanded minimum ARR* and the *estimated ARR* within the group of physicians, a Wilcoxon test was used and medians (*Mdn*) were compared. Box plots were chosen for graphical representation. Demanded minimum and estimated ARR were calculated as:

32%—entered value (x).

Subsequent calculation of NNT resulted from reciprocal ARR. For descriptive statistics, a Mann-Whitney U test and an uneven distribution of the groups, the average ranks (*M*_*Rank*_), U (*U*) and Z statistics (*z*) were displayed. The effect size *r* was based on Cohen with small (*r* = .10), medium (*r* = .30) and large effects (*r* = .50) [40]. The levels of statistical significance were set as follows: *p* < .05 (significant, *), *p* < .01 (very significant, **) and *p* < .001 (highly significant, ***). Due to the low number of fracture occurrence in both groups, no further correlation analysis was carried out. Missing data entry was excluded from quantitative analysis.

## Results

### Sample characteristics

A total of 114 physicians participated in the survey of which 112 physicians (98%) fully completed it. The mean age of physicians was 44.5 (*SD* = 13.8) years. Slightly more female than male physicians participated in the study. Occupational status was evenly distributed between genders. About half of physicians were still in specialty training. Completed specialties mainly were general and internal medicine (**Table 1**).

**Table 1:** Demographic characteristics of physicians.

	*N*	%
	114	100
Age (years)^a^		
18–39	049	44
40–59	042	37
≥ 60	021	19
Gender^b^		
Female	061	54
Male	052	46
Previous fracture^c^		
Yes	004	03
No	110	97
Occupational status		
Employed ambulatory (outpatient)	032	28
Independent ambulatory (outpatient)	040	35
Employed (inpatient)	037	33
Other	005	04
Completed specialist training	0	
None	052	46
General practice/medicine	039	34
Internal medicine	011	10
General & internal medicine	005	04
Other	007	06

Note

^a^ calculated for N = 112, due to missing values.

^b^ calculated for N = 113 due to missing values.

^c^ within the last 12 months.

A total of 140 lay persons participated in the survey of which 130 (93%) fully completed it. The mean age of lay persons was 42.5 (*SD* = 15.3) years. There were substantially more female than male lay persons. Educational level was high. The vast majority had not suffered a fracture within 12 months prior to the study (**Table 2**).

**Table 2:** Demographic characteristics of lay persons.

	*N*	%
	140	100
Age (years)		
18–39	067	48
40–59	037	26
≥ 60	036	26
Gender		
Female	092	66
Male	048	34
Previous fracture^a^		
Yes	005	03
No	135	97
Educational Qualification		
None	003	02
Professional training	029	21
Technical school	015	11
Technical college^b^	027	19
College^c^	060	43
Graduation/doctorate	006	04

Note

^a^ within the last 12 months.

^b^ in Germany: University of applied sciences.

^c^ bachelor/master/diploma/magister/state examination.

### Treatment thresholds

**Fig 2:**
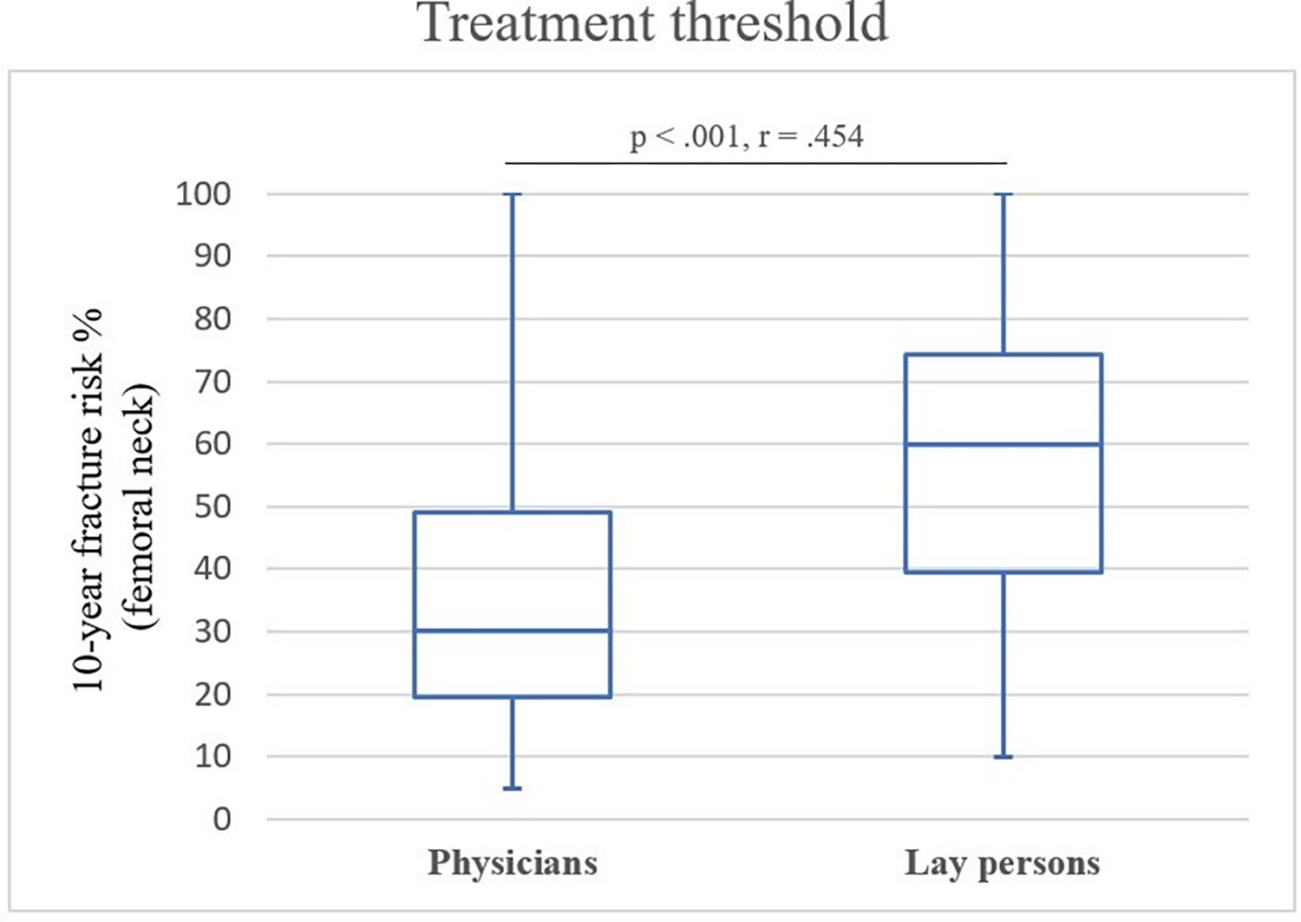
Box plot representation of treatment thresholds of physicians and lay persons. Median, Q1 and Q3 are reported. Whiskers indicate minimum and maximum. Question: “At what individual risk of suffering a femoral neck fracture within 10 years would you prescribe/take bisphosphonates?”.

Valid answers to the question for the treatment threshold were given by *N* = 113 (99%) physicians and *N* = 130 lay persons (93%). The median minimum treatment threshold for prescribing bisphosphonates for physicians was at a 10-year femoral neck fracture risk of 30% (Range 5–100%). The median minimum treatment threshold of lay persons for taking bisphosphonates was at a 10-year risk of femoral neck fracture of 60% (Range 10–100%). Variation of thresholds was high among lay persons with 18% willing to take bisphosphonates already at a 10-year risk of femoral neck fracture ≤ 30%, and 20% of lay persons only at a 10-year risk of ≥ 80%. Median treatment thresholds of both groups were significantly different with a large effect size (*M*_*rank_phy*_ = 87.87, *M*_*rank_lay*_ = 151.67, *IQR*_*phy*_ = 29.7, *IQR*_*lay*_ = 34.7, *U* = 3488.500, *z* = -7.070, *p* < .001, *r* = .454). **Fig 2** displays treatment thresholds as box plots for physicians and lay persons.

### Demanded minimum ARR

**Fig 3:**
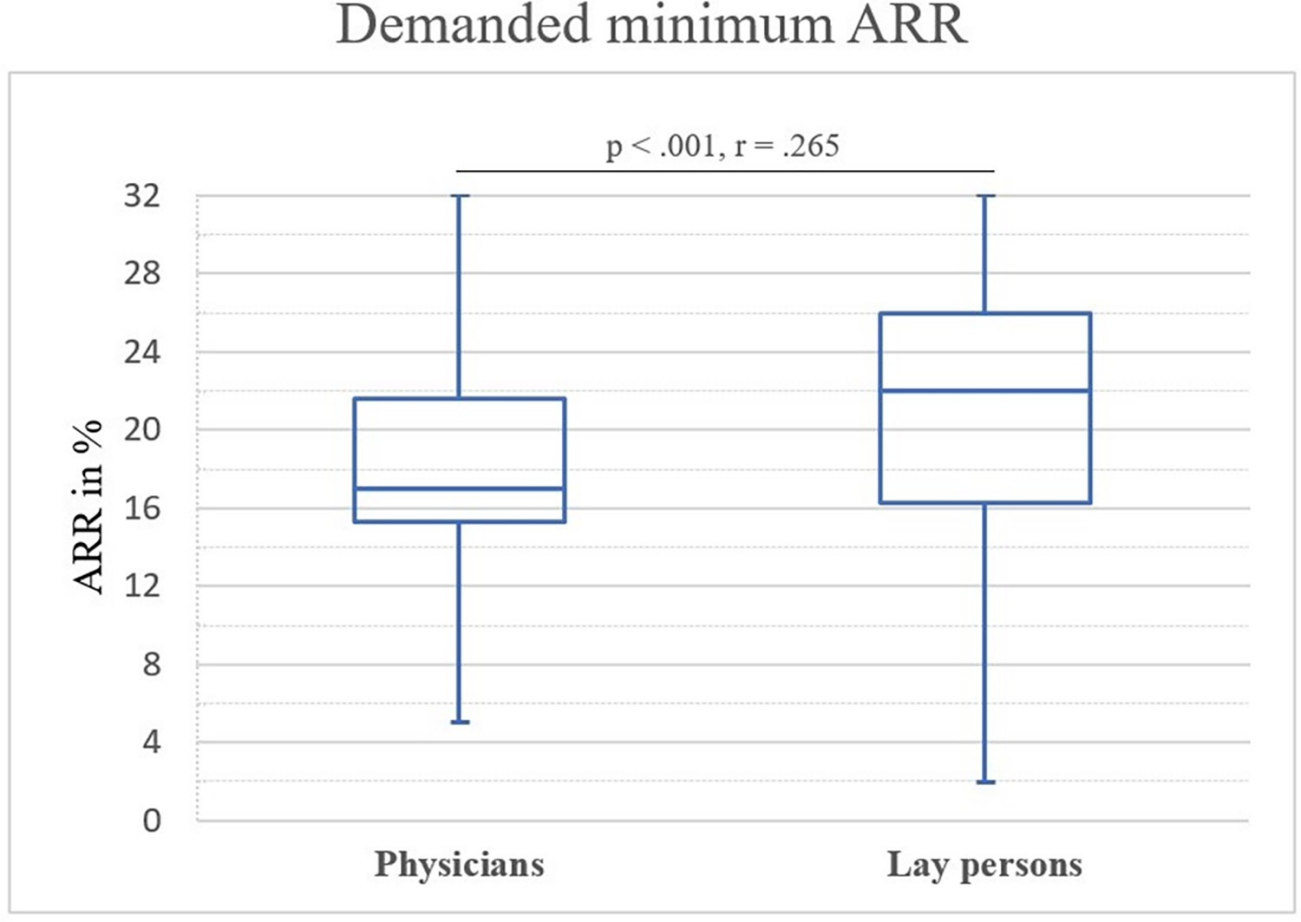
Box plot representation of demanded ARR of physicians and lay persons. Median, Q1 and Q3) are reported. Whiskers indicate minimum and maximum. Question: “What efficacy do you expect of the drug in this case? I expect the drug to reduce the patient’s/my risk from 32% to at least …%.".

Valid answers to demanded ARR were given by 114 physicians (100%) and 135 lay persons (96%). Physicians demanded a median ARR for bisphosphonates of 17% (Range 5–32%, NNT_med_ = 6). An ARR of at least 12% was demanded by 97% of them. Lay persons demanded a median ARR of 22% (Range 2–32%, NNT_med_ = 5). ARR of more than 12% was demanded by 93% of lay persons. Demanded ARRs for both groups were significantly different showing a medium effect size (*M*_*rank_phy*_ = 104.57, *M*_*rank_lay*_ = 142.26, *IQR*_*phy*_ = 6.3, *IQR*_*lay*_ = 9.7, *U* = 5365.500, *z* = -4.181, *p* < .001, *r* = .265). **Fig 3** displays demanded effect sizes in terms of ARR as box plots for physicians and lay persons. A medium correlation between treatment threshold and demanded ARR was present within the physician group (*r* = .339, *p* < .001) but not in the lay person group (*r* = .125, *p* = .163). Physicians with a higher treatment threshold also showed a higher demanded ARR.

### Estimated ARR

**Fig 4:**
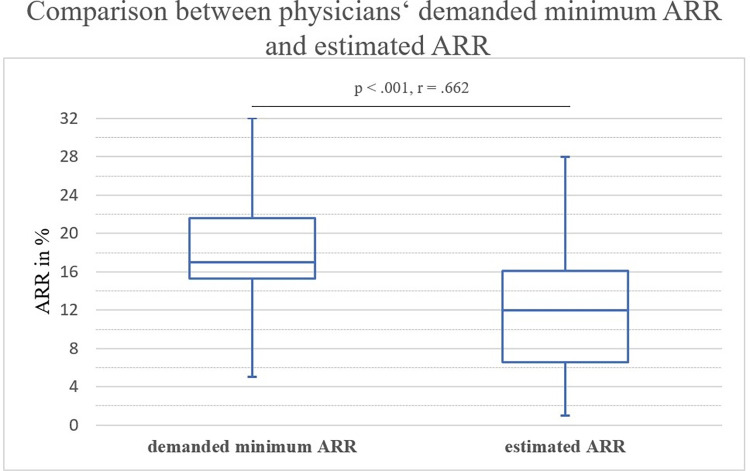
Box plot comparison of demanded minimum ARR and estimated ARR of bisphosphonates of physicians. Median, Q1 and Q3 are reported. Whiskers indicate minimum and maximum.

Valid answers to estimated ARRs were given by 114 physicians. The median estimated ARR of bisphosphonates s by physicians was 12% (Range 1–28%) corresponding to an NNT of 9. **Fig 4** compares physicians minimum demanded ARRs and their estimated real ARRs as box plots. Wilcoxon test revealed that the demanded ARR was significantly higher than the estimated ARR showing a large effect size (*z* = -7.073, *p* < .001, *r* = .662 IQR _demanded minimum ARR_ = 6.3, IQR _estimated ARR_ = 9.5).

#### Influencing variables

In the physician group there was no correlation between gender, age groups, specialist training or occupational status on the one side and treatment threshold, expected and estimated ARR on the other. In the lay person group there was no correlation between gender, age groups, educational status and treatment threshold or demanded minimum ARR.

## Discussion

When presented with a patient scenario lay persons were willing to accept a twice as high median 10-year risk treatment threshold of femoral neck fracture and demanded a higher risk reduction from bisphosphonates than physicians. Physicians demanded a higher risk reduction from bisphosphonates than they would estimate the real benefit of these medications. Both lay persons and physicians demanded bisphosphonates to be more beneficial than they actually are according to evidence. Treatment thresholds of 10-year fracture risk for all osteoporotic fractures as set by the guidelines are between 14% and 20%. The treatment threshold expressed by the German physicians for femoral fractures only was identical to the treatment threshold for hip and spine fractures underlying the German guideline recommendations for osteoporosis [1]. However, we had asked for the minimum risk threshold in a distinct patient case and only for femoral fractures while guidelines present their thresholds for a risk based on some or all major fracture types. The threshold for femoral fractures only is not given in the German guideline but given in the NOF guideline as ≥ 3% for ten years. As the fracture risk for femoral fractures generally is much lower than the risk for all major osteoporotic fractures combined, physicians can be expected to set the threshold for all major osteoporotic fractures much higher than the guidelines recommend.

The actual ARR of bisphosphonates for femoral neck fracture reduction in the studies reaches about .57% to 1% over three years [22, 41]. There is evidence for the efficacy of bisphosphonates no longer than five years [1]. The 10-year baseline risk for the patient in our case vignette was with 32% much higher than the mean baseline risk in the studies. To our knowledge, studies with patients with a 10-year baseline risk > 20% do not exist. It is difficult to estimate, whether the effect of bisphosphonates on femoral fractures rises, equals or diminishes with higher fracture risk in comparison to existing studies. With rising age of the patient, osteoporosis and non-skeletal risk factors both inevitably increase. Patients with very high fracture risk might have very low BMD or very high non-skeletal risk factors like frailty and falls. The latter will be the most common reason in the oldest old for whom bisphosphonates seem to be less effective for preventing these fractures, if they are effective at all [23]. Thus, depending on the factors contributing to this high risk for femoral fractures, the ARR for the patient in the vignette might be higher than the .57% to 1% if depending on severe osteoporosis or even lower or zero if depending on non-skeletal risk factors. Anyway, the older the patient gets, the less meaningful will it be to calculate a 10-year fracture risk. There obviously is a mismatch between 10-year fracture risk estimations and the much shorter observation time in studies of antiosteoporotics which usually is three years. If evidence can’t tell us how much 10-year fracture risk can be decreased by antiosteoporotic medication and with rising age increasing the chance of the patient’s death due to age itself, it might not make much sense to operate with such a measure. Assuming fracture risk reduction lasts about five years [42]—the theoretical cumulative maximum effect in risk reduction of femoral neck fractures would be approximately 3–3.5% over five years. 10-year extrapolation would reveal a best estimate maximum benefit of 6–7% ARR and seems highly problematic as age and ageing are only limited proxies for functional health. Fracture risk will mostly rise steeply with rising grades of frailty. However, frailty at the same time is a good proxy for approaching death, resulting in the trap that with rising fracture risk there will be less time to experience the benefits of preventive antiosteoporotic medication. The median minimum efficacy demanded by lay-persons being 22% was far above this insecure best estimate which also accounts for the 17% ARR physicians demanded. Most physicians estimated ARR to be greater than 5%, which would include this extrapolated maximum benefit of 6–7% ARR. However, the median estimated benefit was 12% thereby doubling the “real” ES. This generates a rather contradictory situation for physicians. They overestimated the effects of bisphosphonates and demanded even higher ES in order to be willing to prescribe them. A possible explanation for this phenomenon might be that physicians are not familiar and normally don’t think in the way we asked them. Taking rational decisions based on numbers of ES, though probably the better way to take decisions and proposed as such by the current medical paradigm evidence-based medicine (EBM), might correspond only little to the way physicians actually think and decide [43]. But even if this kind of rational thinking might not be exacted from physicians who in Germany still mostly have never been trained in EBM, it should very well be exacted by authors of guidelines claiming the evidence-based label. These findings urge physicians and patients to learn about and communicate best estimates of ES of antiosteoporotic therapy in order to avoid overtreatment.

### Comparison to other studies

Other studies revealed similar results thereby supporting our findings. Steel et al. asked the general population and different groups of medical professionals or physicians for distinct NNT categories to take antihypertensive medication over five years in order to prevent one death [13]. Specialists accepted the highest median NNT = 100, followed by general practitioners NNT = 50, health professionals and general population NNT = 33. Douglas et al. conducted a survey on patients referred for BMD measurement and physicians managing osteoporosis [12]. The treatment threshold of 10-year absolute risk for *major osteoporotic or hip fracture* justifying intervention was five times higher (50%) for patients than for their physicians (10%), supporting the hypothesis of different risk perception. In contrast to our study, Douglas et al. asked patients not lay persons and the majority of patients was over 60 years of age. Their sample consisted mostly of general practitioners as opposed to only a third of them in our study. Still, our study shows similar results in the discrepancy of intervention thresholds between lay persons/patients and physicians. We were unable to find other studies asking for minimum ES or estimations of ES of antiosteoporotic drugs to compare our results with. But interestingly, a systematic review identified several factors influencing a patient’s anti-osteoporotic medication adherence by presenting five factors physicians should consider in their daily practice (individual condition, patient-related factors, therapy-related factors, health-system and socio-economic factors) [44], emphasizing the need to practice EBM and shared-decision making in osteoporosis. This creates a rather paradoxical situation. On the one hand, guidelines often insufficiently communicate ES or MCID to the very same physicians who should apply them or deviate from them in an evidence-based way [9]. Even if ES or MCID would be presented in guidelines, German physicians themselves identify a “lack of EBM skills” as barrier to seek for and evaluate information in the first place [45]. If an ES or MCID is neither sufficiently reported, nor comprehensibly presented to the physician, nor appropriately understood by physicians, it remains doubtful if it can be explained to the patient in the shared-decision making process.

### Limitations

The snowball distribution system of the email with the link to the survey resulted in an unknown number of recipients. Selection bias due to differences in motivation to participate cannot be ruled out. Therefore, our convenience sample cannot be regarded as representative. The biggest limitation of our study probably is the fact that our survey did not include enough older lay persons or patients and no orthopedic surgeons. The German health care system allows patients to directly consult specialists like orthopedic surgeons in ambulatory care. Orthopedic surgeons probably start prescribing antiosteoporotic medication in the majority of cases in Germany. The reason for the important lack of these two most important groups was that in the pretest of the questionnaire we learned that old people had major difficulties understanding what we were asking for. Furthermore, we were unable to recruit enough orthopedic surgeons despite various attempts with the snowball distribution system in our sample. Also, our sample contained an increased proportion of lay persons with a university degree not corresponding to the German population average [46]. The questions in the two questionnaires were not randomized, making positioning effects in the responses possible. Framing effects and bias due to different wording in the information texts for physicians and lay persons cannot be ruled out. Lastly, the survey’s given choice of the time span over ten years to take a medication could reduce the willingness to take or prescribe medication in the first place. Another weakness might be seen in the fact that we did not ask for the role of harms which should be part of any rational decision-making process.

## Conclusions

Our results show a relevant gap of intervention thresholds and demanded ES between physicians and lay persons in order to be willing to prescribe or take antiosteoporotic medication. In addition, physicians estimated the actual ARR of antiosteoporotic medication to be even lower than they themselves demanded to be the minimum ES in order to prescribe them. This study adds, with some limitations, to the still sparse body of evidence finding out about this patient-physician gap for osteoporosis and fragility fractures as for probably most other clinical situations of decision making. As a precondition for shared decision making, guidelines should provide their readers with understandable ES of benefits and harms in order to allow patients and physicians taking individual decisions.

## Supporting information

S1 Appendix STROBE checklist. (DOCX)

S2 Appendix CHERRIES checklist. (DOCX)

S3 Appendix Questionnaire for physicians and lay persons. (DOCX)

S4 Appendix Raw data. (ZIP)
