# Supplementary material for: Treatment thresholds and minimal clinically important effect sizes of antiosteoporotic medication–Survey among physicians and lay persons in Germany
Source: PLoS One. 2022 Aug 11;17(8):e0272985. doi: 10.1371/journal.pone.0272985 (PMC9371311; doi:10.1371/journal.pone.0272985)
Supplement: S3 Appendix — (DOCX) [file pone.0272985.s003.docx]

**Appendix S3 – questionnaire for physicians and lay persons**

A. Physicians

**1.1. What is your gender?**

- male
 - female
 - diverse

**1.2. How old are you?**

*[text entry, two digits]*

**1.3. What is your specialist training/specialist title?**

*[text entry]*

1.4. **What is your current occupational status?**

- employed ambulatory (outpatient)
 - independent ambulatory (outpatient)
 - employed stationary (inpatient)
 - other

**2. Information**: Fracture risk increases with age and with decreasing bone density. Antiosteoporotic medication (e.g. bisphosphonates) reduce the fracture risk in osteoporosis, but cannot completely rule out a fracture. Possible side effects of these drugs can among others include bone, muscle and joint pain or hair loss (in around 100 out of 1,000 people) and allergic reactions or diseases of the jawbone (in up to 1 out of 1,000 people).

**2.1. At what individual risk of suffering a femoral neck fracture within 10 years would you prescribe bisphosphonates?** *[Please move the slider to the lowest percentage that you can still accept. Slider from 0 % (left) – 100 % (right), starting position at 0%]*

Please imagine the following situation: Your 78-year-old patient was diagnosed with osteoporosis. She had no prior fracture due to this condition. Now assume, that the risk of suffering a femoral neck fracture within 10 years is 32% for this patient without medication. This means that 32 out of 100 patients with these conditions will suffer such a fracture. Now let’s presume, you decide to prescribe a drug to reduce the fracture risk.

**2.2. What efficacy do you expect of the drug in this case?**
"*I expect the drug to reduce the patient's risk from 32 % to at least (x) %." [Please move the slider to the highest percentage that you can still accept. Slider from 32 % (right) – 0 % (left), starting position 32%]*

**2.3. How do you estimate the actual benefit of therapy with bisphosphonates in the aforementioned case? (78 years; female; BMI 25.7; T-Score: -3.0; no other risk factors)**

*"The patient's risk of suffering a femoral neck fracture within 10 years is reduced from 32% to ...% by regularly taking a bisphosphonate." Slider from 32 % (right) – 0 % (left), starting position 32%]*

4. Did you suffer from a fracture within the last 12 months?
 - yes
 - no

B. Lay persons

**1.1. What is your gender?**

- male
 - female
 - diverse

**1.2. How old are you?**

*[text entry, two digits]*

**2. Information:** Fracture risk increases with age and with decreasing bone density. If this density falls below a certain value, this is called osteoporosis. Fractures may not only lead to pain for the individual affected. For example, in case of a femoral neck fracture, a hip replacement often has to be implanted, which is associated with hospitalization. Additionally, complications such as healing disorders, reduced mobility or chronic pain can occur. Certain medications (e.g. bisphosphonates) reduce the fracture risk in osteoporosis, but they also can not completely rule out a fracture. Possible side effects of these drugs can among others include bone, muscle and joint pain or hair loss (in around 100 out of 1,000 people) and allergic reactions or diseases of the jawbone (in up to 1 out of 1,000 people).

**2.1. At what personal risk of suffering a femoral neck fracture within 10 years would you take a drug?** *[Please move the slider to the lowest percentage that you can still accept. Slider from 0 % (left) – 100 % (right), starting position at 0%]*

Please imagine the following situation: You are 78 years old and you have been diagnosed with osteoporosis. You have not noticed any the disease yourself and have not yet suffered a fracture that could be associated with osteoporosis. Now assume your risk of suffering a femoral neck fracture within 10 years without medication is 32%. This means that 32 out of 100 patients with your conditions will suffer such a fracture. Let’s presume you decide to take a medication to reduce your risk.

**2.2. What efficacy do you expect of the drug in this case?**"I expect the drug to reduce my risk from 32 % to at least (x) *%." [Please move the slider to the highest percentage that you can still accept. Slider from 32 % (right) – 0 % (left), starting position 32%]*

**3. Did you suffer from a fracture within the last 12 months?**
 - yes
 - no

**4. What is your highest educational qualification?**

- None
- Professional training
- Technical school
- Technical college

- College
- Graduation/doctorate
